# Clonality and antimicrobial susceptibility of methicillin-resistant *Staphylococcus aureus* at the University Hospital Zurich, Switzerland between 2012 and 2014

**DOI:** 10.1186/s12941-015-0075-3

**Published:** 2015-03-19

**Authors:** Kati Seidl, Nadja Leimer, Miguel Palheiros Marques, Alexandra Furrer, Anne Holzmann-Bürgel, Gabriela Senn, Reinhard Zbinden, Annelies S Zinkernagel

**Affiliations:** Division of Infectious Diseases and Hospital Epidemiology, University Hospital Zurich, University of Zurich, Rämistr. 100, RAE U, 8091 Zurich, Switzerland; Institute of Medical Microbiology, University of Zurich, Zurich, Switzerland

**Keywords:** MRSA, Epidemiology, Antibiotic susceptibility, Molecular typing

## Abstract

**Background:**

Methicillin-resistant *Staphylococcus aureus* (MRSA) is a global epidemic threat. The aim of this study was to determine which globally known MRSA lineages are currently present at our tertiary care hospital in Switzerland, a hospital with low MRSA prevalence. In light of the increasing prevalence of multi drug resistance including vancomycin resistance we also assessed antibiotic susceptibilities.

**Methods:**

The 146 MRSA strains collected over two years (March 2012 until February 2014) at the University Hospital Zurich, Switzerland, were analyzed by PFGE analysis of SmaI digests in combination with *spa*-typing. In addition, representative isolates were analyzed by multi locus sequence typing (MLST). Susceptibilities to eight antibiotics were assessed using the Kirby-Bauer disc diffusion method.

**Results:**

Isolates showed resistance to erythromycin (48%), ciprofloxacin (43%), clindamycin (31%), tetracycline (22%), and gentamicin (16%). All isolates were susceptible to vancomycin, 95% were susceptible to sulfamethoxazole/trimethoprim and rifampicin, respectively. PFGE analysis revealed 22 different patterns, with four major patterns that accounted for 53.4% of all MRSA isolates, and seven sporadic patterns. *Spa* typing revealed 50 different *spa* types with the predominant types being t008 (14%), t002 (10%), and t127 (9%). 82% of the MRSA isolates could be assigned to six clonal complexes (CCs) namely CC1 (10%), CC5 (23%), CC8 (18%), CC22 (17%), CC30 (11%), and CC45 (3%) based on *spa*-types, PFGE patterns, and MLST. Two isolates could not be typed by either PFGE analysis or *spa*-typing and three isolates had *spa*-types that have not yet been described.

**Conclusions:**

The combination of the two typing methods was more discriminatory as compared to the use of a single method. Several of the lineages that are predominant in Europe are present in our hospital. Resistances to antibiotics have decreased in comparison to a study conducted between 2004 and 2006.

**Electronic supplementary material:**

The online version of this article (doi:10.1186/s12941-015-0075-3) contains supplementary material, which is available to authorized users.

## Background

Methicillin-resistant *Staphylococcus aureus* (MRSA) is one of the major causes of healthcare-associated infections worldwide [1]. In addition, the increasing prevalence of multi drug resistance including vancomycin resistance emphasizes the importance of infection control measures such as MRSA typing. There are considerable variations in the prevalence of MRSA according to geographic area and rates reach over 50% in some regions of the world (Reviewed by Stefani *et al.* 2012 [2]). Most MRSA strains belong to a few distinct pandemic lineages. The current terminology to describe *S. aureus* lineages is based on the clonal complexes (CCs) identified by multilocus sequence typing (MLST). MLST involves the sequencing of seven housekeeping genes and each unique allelic profile is assigned a sequence type (ST) [3]. Clonal complexes are defined as groups of STs in which every ST shares at least five of seven identical alleles with at least one other ST in the group [4]. Even though numerous studies have used whole genome sequencing to explore the local and global dissemination of distinct lineages recently (for example [5]), MLST is still considered the “gold standard” of typing. But since for MLST seven house-keeping genes have to be sequenced it is expensive and time-consuming. Therefore, many epidemiological studies have used *spa*-typing, which includes the sequencing of the polymorphic repeat region of protein A [6]. In the meantime, most CCs can be predicted from *spa*-types using previous publications (for example [7]) or the ridom *spa*-server (http://spa.ridom.de/spatypes.shtml [8]). Other methods currently used to type MRSA include SCC*mec* typing, or multilocus variable-number tandem repeats analysis (MLVA), and were recently reviewed [2]. SCC*mec* typing uses a defined nomenclature, but there are several typing and subtyping schemes that are not harmonized, and the discriminatory power of this method is limited. Even though MLVA is rapid, high-throughput and has high discriminatory power, no standard methodology or nomenclature has been defined, which makes this method less suitable for assessing long-term and global epidemiology [9].

Especially before the introduction of sequence-based approaches pulsed field gel electrophoresis (PFGE) used to be the “gold standard” of MRSA typing [10]. PFGE analysis is very convenient and has high discriminatory power. PFGE is still widely used for short-term, local epidemiology, and to identify outbreaks. However, it is less suited for studying long-term and global epidemiology since it does not permit to compare between centers. This is reflected by the fact that PFGE patterns (e.g. numbers) used at our hospital are only valid for strains typed at our hospital. In addition, it is challenging to compare PFGE data over long time periods, since several variables (i.e. devices and personnel) might change over time and since PFGE analysis is, at least to a certain degree, a matter of subjective interpretation, i.e. whether a weak band is assessed or not.

All MRSA isolates collected at our hospital, a tertiary care hospital in Switzerland with low MRSA prevalence (3-6%, [11]), have been analyzed by PFGE since 1992 within the scope of our local isolation management. The objectives of the current study were to combine the PFGE data with an additional typing method (*spa*-typing), and to determine which of the globally known clones were found at our hospital during a two-year study period. MLST served as reference. In addition, antibiotic susceptibilities of the MRSA strains were assessed.

## Methods

### Bacterial strains

All consecutive, non-duplicate MRSA strains collected at the University Hospital Zurich, Switzerland between March 2012 and February 2014 were included in the study. Isolates included MRSA from patients with infections (including three bacteremias) as well as from colonized individuals. Several reference strains were obtained from the Network on Antimicrobial Resistance in *Staphylococcus aureus* (NARSA) and included USA300-114 (ST8), USA100 (ST5), USA1100 (ST30), USA400 (ST1), USA700 (ST72), and USA500 (ST8). EMRSA-15 (ST22) was a kind gift of Dr. Patrice François (Geneva, Switzerland). Strain CHE482 (ST45) has been previously described [12].

### Methicillin resistance detection

Isolates were identified as *S. aureus* by StaphAureux tests (Remel, Kent, UK). Methicillin resistance was detected by testing cefoxitin resistance using the Kirby-Bauer disc diffusion method [13] and/or by detection of the penicillin binding protein 2a (PBP2a) using the MRSA Screen kit (Denka Seiken Co., Ltd., Tokyo, Japan).

### Susceptibility testing

Resistance levels for ciprofloxacin, clindamycin, erythromycin, rifampicin, tetracycline, gentamicin, sulfamethoxazole/trimethoprim, and vancomycin were determined by disk diffusion according to EUCAST guidelines [13]. To compare susceptibility patterns of the current strain collection with a collection from a previous study that has been conducted in 2007 [11], susceptibilities were also interpreted using CLSI guidelines [14]. Intermediate resistance and inducible resistance were assumed to be resistant.

### Molecular typing

PFGE typing was done with SmaI digests according to standard protocols and analyzed using the BioNumerics software package (Applied Maths, Belgium). The dice coefficient was used with 1.25% optimization and 1% tolerance to calculate similarities between PFGE patterns. Isolates with >80% identity were considered identical. Isolates with similar PFGE patterns (70% identity) were assigned with letters (e.g. 21a). Numbers were assigned to isolates that occurred at least three times in our laboratory including isolates from other hospitals and isolates that were collected before 2012. Therefore, some of the PFGE patterns only occurred once in this study but have a number. *Spa* sequence typing of the polymorphic repeat regions of protein A [6] was performed using the Ridom StaphType *spa*-sequencing protocol and the *spa*Typer (http://spatyper.fortinbras.us/). Multi-locus sequence typing (MLST) of representative isolates was performed as described [3] and sequence types (STs) were derived using the MLST database (http://www.mlst.net).

## Results and discussion

### Resistance patterns

Of the 146 isolates 48% showed resistance (or intermediate resistance) to erythromycin, 43% to ciprofloxacin, 31% to clindamycin (of which 14% were inducible), 22% to tetracycline, and 16% to gentamicin (Table 1). All of the 146 isolates were susceptible to vancomycin, and 95% of isolates were susceptible to sulfamethoxazole/trimethoprim and rifampicin, respectively (Table 1). Thirty seven isolates (25%) were susceptible to all antibiotics other than oxacillin. There was a decrease in resistances to ciprofloxacin, erythromycin, gentamicin, rifampicin, and tetracycline as compared to MRSA strains collected between 2004 and 2006 at our hospital [11]. In this previous study, 67% of isolates were resistant to ciprofloxacin (25% more than in the current study), 57% to erythromycin (9% more), 34% to tetracycline (12% more), 22% to gentamicin (7% more), and 11% to rifampicin (6% more). No information was available about the resistance towards sulfamethoxazole/trimethoprim or whether the clindamycin-resistant strains exhibited inducible resistance. Because some of the changes in resistances might be attributable to the change in guidelines from CLSI to EUCAST we also analyzed antibiotic susceptibilities of the current collection using the CLSI guidelines which had been used in the previous study [14]. Decreases were identical for ciprofloxacin, erythromycin, and tetracycline when applying CLSI guidelines. Decreases in resistance to gentamicin and rifampicin were slightly higher (9% and 7% less resistant strains in the current vs. the previous study, respectively).

**Table 1:** **Resistance phenotypes of MRSA**

**Resistance profile**	**No. (%) of isolates**
***Individual antibiotics***	
Erythromycin	**70 (47.9)**
Ciprofloxacin	**62 (42.5)**
Clindamycin (including inducible resistance)	**45 (30.8)**
(Inducible Clindamycin resistance)	20 (13.7))
Tetracycline	**32 (21.9)**
Gentamicin	**23 (15.6)**
Sulfamethoxazole/trimethoprim	**8 (5.4)**
Rifampicin	**7 (4.8)**
Vancomycin	**0 (0)**
***Multiresistance***	
**Oxacillin + 1 antibiotic**	**42 (28.8)**
Ciprofloxacin	16 (11.0)
Erythromycin	10 (6.8)
Tetracycline	9 (6.1)
Gentamycin	6 (4.1)
Sulfamethoxazole/trimethoprim	1 (0.7)
**Oxacillin + 2 antibiotics**	**22 (15.1)**
Ciprofloxacin + erythromycin	7 (4.8)
Clindamycin + erythromycin	6 (4.1)
Erythromycin + tetracycline	3 (2.1)
Other combinations*	6 (4.1)
**Oxacillin + 3 antibiotics**	**29 (19.7)**
Ciprofloxacin + clindamycin + erythromycin	16 (11.0)
Ciprofloxacin + erythromycin + tetracyclin	7 (4.8)
Other combinations*	6 (4.1)
**Oxacillin + 4 antibiotics**	**11 (7.5)**
Ciprofloxacin + clindamycin + erythromycin + gentamicin	6 (4.1)
Other combinations*	5 (3.5)
Oxacillin + 5 antibiotics*	**1 (0.7)**
Oxacillin + 6 antibiotics*	**4 (2.7)**
Ciprofloxacin + clindamycin + erythromycin + tetracyclin + rifampicin + gentamicin	3 (2.1)

*Combinations that occurred less than three times are not specified. Bold numbers indicate the total number of each subgroup.

Multiresistance to one, two, three, four, five, and six antibiotics in addition to oxacillin was observed for 29%, 15%, 20%, 8%, 1%, and 3%, of the isolates, respectively (Table 1). The most prominent combinations of resistances are shown in Table 1. Together, ~30% of the MRSA strains isolated were resistant to at least four antimicrobial agents (including oxacillin), emphasizing that antibiotic resistance remains a problem. These findings underline the importance of infection control measures.

### PFGE analyses

**Figure 1:**
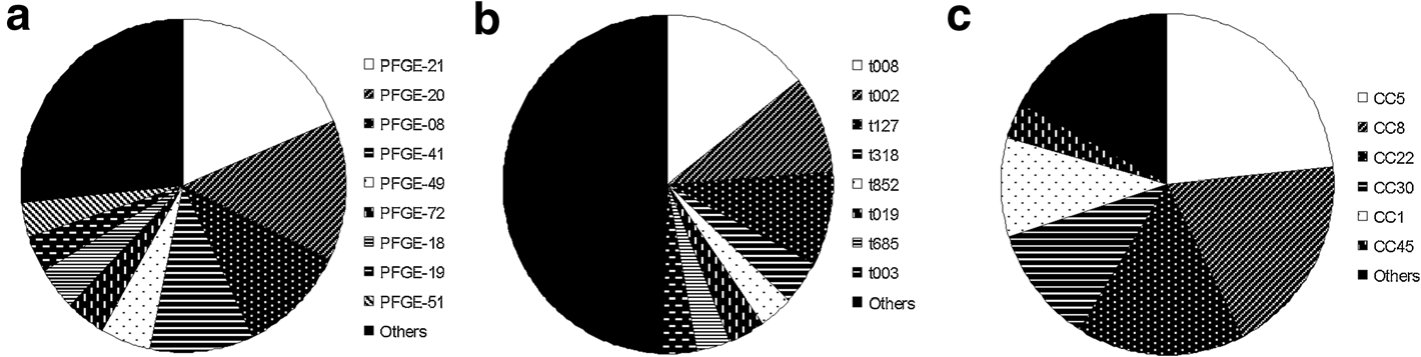
**Genetic background of MRSA.** Frequencies of PFGE types **(a)**, *spa*-types **(b)**, and predicted clonal complexes **(c)**. CC, clonal complex.

Of the 146 isolates 136 isolates were grouped into 22 PFGE patterns. The four most frequent PFGE patterns were PFGE-21 (*n* = 27, 19%), PFGE-20 (*n* = 20, 14%), PFGE-08 (*n* = 16, 11%) and PFGE-41 (*n* = 15, 10%), and together made up more than 50% of all isolates (Figure 1a). Nine isolates had a pattern that had never occurred in our laboratory and were therefore not assigned a number. One isolate (MRSA3687) could not be typed by PFGE analysis (Additional file 1: Table S1).

### *Spa*-types

The 146 isolates had 50 distinct *spa*-types. The three most common *spa*-types were t008 (*n* = 21, 14%), t002 (*n* = 14, 10%), and t127 (*n* = 13, 9%) and accounted for 33% of all isolates (Figure 1b). All other *spa*-types occurred less than ten times and 47% of all isolates had a *spa*-type that occurred less than three times (Figure 1b). One of the 146 isolates (MRSA3984) was not *spa*-typable (no PCR product for *spa*). Three isolates had *spa*-types not described so far: MRSA3903 (T1-J1-J1-*-J1-N1-F2-M1-N1-F2-M1-O1-O1-K1-R1) had a repeat that differed in one nucleotide from repeat r13 (E1). MRSA 3771 had a new combination of *spa*-repeats (T1-N1-F2-M1-O1-M1-O1-O1-R1) as well as MRSA3670 (U1-J2-G1-M1-K1-M1-K1-K1-P1-N1-S1-G1, Additional file 1: Table S1).

The two most common *spa*-types found in this study (t008 and t002) were among the most frequent *spa*-types in MRSA blood stream isolates collected all over Europe from 2006 to 2007 [15]. In contrast to this previous study, our MRSA strains were derived from various infections including three blood stream isolates (one of which was of t008) as well as from colonized patients. We thus provide information on both invasive and colonizing MRSA strains. In contrast, we have no information whether the European blood stream isolates represent the MRSA circulating in the population. *Spa*-type t127, our third most common *spa*-type, was only sporadically identified in the European blood stream isolates [15]. However, this *spa*-type has been identified as a common MRSA lineage in pigs in Europe, mainly in Italy [16]. Several common *spa*-types found in European blood stream isolates e.g. t032 (14.5%) or t041 (7.4%) only occurred sporadically in our hospital (t032 (5%)) or were absent (t041). In contrast, t041 was the local epidemic *spa*-type of MRSA isolates (including invasive and colonizing isolates) at the University Hospital Basel, Switzerland between 2000 and 2005 [17], another low-prevalence hospital less than 100 kilometers away from our hospital.

### Correlation between PFGE analyses and *spa*-typing

**Figure 2:**
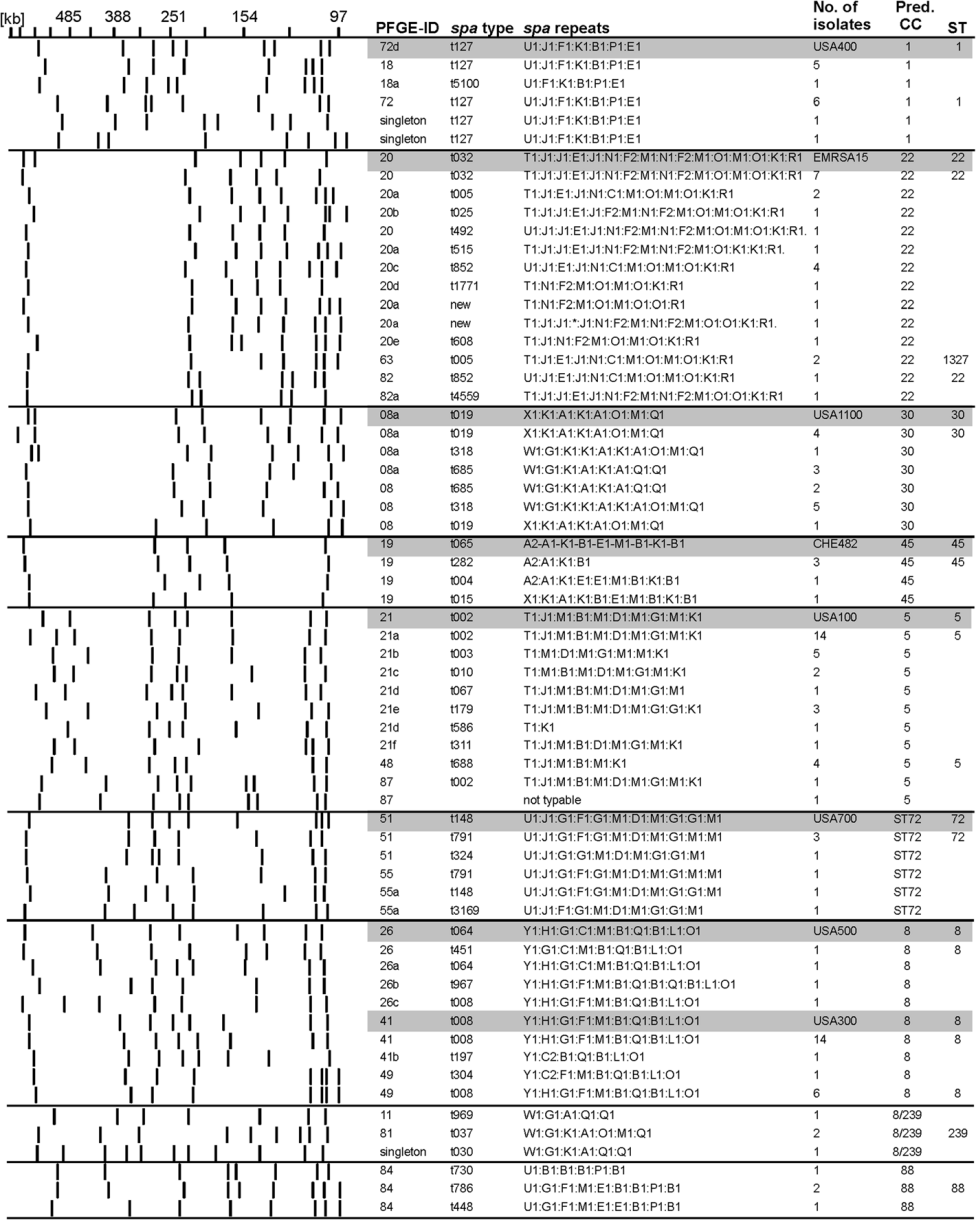
**Correlation between PFGE analyses and** ***spa*** **-typing.** Genetic relationships between the 146 MRSA isolates, correlations between PFGE typing and *spa*-typing (*spa*). Strains presented were sorted according to PFGE patterns and similarities of *spa*-types. Shown are strains of clonal complexes (CC) that occurred at least four times. Reference strains are marked with grey boxes. ST, Sequence type.

Results of PFGE analyses and *spa*-typing are shown in Figure 2. All isolates were typable by at least one of the two methods. Two isolates could not be typed by either PFGE analysis or *spa*-typing. Both phenomena have been previously described and were reported to be due to methylation of the SmaI restriction site as well as due to mutations of the protein A gene *spa*, respectively [18,19].

In most of the cases, isolates with identical PFGE patterns had identical or related *spa*-types. Only two isolates (MRSA3548 and MRSA3619) which were both of *spa*-type t127 were both singletons with low similarity (~50%) to the other t127 isolates (Figure 2).

Different PFGE patterns shared common *spa*-types (e.g. PFGE-41 and 49 both included *spa*-type t008). *Vice versa,* several strains with different *spa*-types shared the same PFGE patterns (e.g. PFGE-21 included seven different *spa*-types, Figure 2). These findings emphasize that the combination of both methods is more discriminatory as compared to using one method only as has been described previously [20].

### Clonal complex 5

Thirty-four isolates were of *spa*-types t002, t003, t010, t067, t179 or t688 that corresponded to CC5 [7,8,21,22]. These 34 isolates were grouped into three related PFGE-patterns (PFGE-21, 48, and 87 (Figure 2)), one of which (PFGE-21) was identical to USA100 (ST5, CC5). MLST of representative PFGE-21/t002 and PFGE-48/t688 isolates confirmed that both were of ST5, CC5. ST5 was one of the predominant MRSA clones among blood culture isolates in Europe [15].

### Clonal complex 8 and related lineages

Twenty-eight isolates were grouped into CC8 based on their *spa*-types, which were t008, t064, t304, t451, t967 [22,23]. These isolates displayed the PFGE patterns 41, 49, and 26 (Figure 2). PFGE-41 had previously been identified as USA300 (ST8, CC8) [24]. This lineage is the most prevalent MRSA in the United States of America (USA) and a global epidemic threat (Reviewed by Nimmo 2012 [25]). Special care must be taken to avoid the spread of this particular clone. The PFGE-49 pattern has been previously shown to be ST8 [24]. PFGE-26 was identical to USA500 (ST8, CC8), and we confirmed ST8 by MLST.

Isolates that were related to CC8, but were grouped separately as previously suggested [26], included four isolates that were assigned to ST239 (CC8/239) based on their *spa*-types t030, t037 and t969 [26]. MLST of one of these isolates confirmed that they were of ST239. Seven isolates were of *spa*-types t148, t324 and t791, t3169, which are predictive of ST72 and also related to CC8 [26]. These isolates belonged to PFGE-51 (*n* = 4) or PFGE-55 (*n* = 3). PFGE-51 exhibited >70% identity with USA700 (ST72) and MLST confirmed that PFGE-51 was ST72.

### Clonal complex 22

Twenty-four isolates were of *spa*-types t005, t032, t492, t515, t852, t1771, t4325, t4559 (Table 1), which was predictive of CC22 [8,27]. Twenty of these isolates (PFGE-20) were identical to EMRSA-15 (ST-22, CC22) in terms of PFGE-restriction patterns. MLST of a representative isolate confirmed that these isolates were of ST22. EMRSA-15 (ST22) was also widely distributed among MRSA blood stream isolates in Europe [15]. The other four isolates were of PFGE-82, which was also of ST22 and PFGE-63, which was of ST1327, a sequence type that differs in one allele (*pta*) from ST22 (Figure 2).

### Clonal complex 30

Sixteen isolates had *spa*-types t019, t318 and t685, which are indicative of CC30 [22]. All of these isolates had the PFGE-08 pattern, which exhibited at least 80% identity to USA1100 (ST30, CC30). MLST of a representative PFGE-08 isolate confirmed that it was of ST30.

### Clonal complex 1

Fourteen isolates were of *spa*-type t127 and the related *spa*-type t5100 which was assigned to CC1 [22]. These isolates had PFGE-18 (*n* = 6) and PFGE-72 (*n* = 6) patterns and two isolates were singletons. PFGE-72 exhibited >80% similarity to USA400 (ST1, CC1). MLST of a PFGE-72/t127 isolate confirmed that it was of ST1, confirming its grouping into CC1 (Figure 2).

### Clonal complex 45

Five isolates were of *spa*-types t004, t015 or t282, which are predictive of CC45 [8], a lineage that is common in Europe [2]. These isolates were of PFGE-19, which is identical to the Swiss drug clone CHE482-ST45 [12]. MLST of a representative PFGE-19 isolate confirmed that it was ST45. The Swiss drug clone is a community acquired MRSA (CA-MRSA) clone, which was spread among injection drug users, was predominant at our hospital between 1998 and 2004 [11], and still occurs sporadically.

### Summary of clonal complexes

Four isolates were of *spa*-types t448, t730 and t784, being indicative of CC88 [28]. MLST of one of these isolates confirmed that it was ST88. The remaining thirteen isolates were of diverse genetic backgrounds (Additional file 1: Table S1). Together, 82% of the MRSA isolates could be grouped into the six clonal complexes (CCs): CC1 (10%), CC5 (23%), CC8 (18%), CC22 (17%), CC30 (11%), and CC45 (3%) based on their PFGE pattern, *spa* types, MLST and previously published literature (Figure 1c).

### Correlation between genetic background and antibiotic sucseptibility

Additional file 1: Table S1 shows the antibiograms of the individual MRSA strains. While some clones, such as PFGE-21a/t002 or PFGE-41/t008 showed high heterogeneity in their antibiograms, other clones had homogenous resistance patterns. *Spa*/PFGE-types that shared identical antibiograms included t003 isolates (*n* = 5, PFGE-21b, CC5), t688 isolates (*n* = 4, PFGE-48, CC5), t685 isolates (*n* = 5, PFGE-08 and PFGE-08a, CC30), t005 (*n* = 4, PFGE-20 and PFGE-63, CC22), t282 isolates (*n* = 3, PFGE-19, CC45), and PFGE-51 isolates (*n* = 4, t791 or t324, ST72) (Additional file 1: Table S1). Interestingly, all four ST239 isolates were resistant to six antibiotics in addition to oxacillin, which is in accordance with previous findings, in which ST239 has been described as multi resistant [5].

## Conclusions

In summary, we have demonstrated that i) several of the lineages that are predominant in Europe are present in our hospital, ii) the combination of PFGE and *spa*-typing is more discriminatory as compared to using a single method only and will allow to better monitor and recognize changes in MRSA epidemiology over time, iii) clonal complex predictions based on PFGE and *spa*-typing could be confirmed by MLST, and iv) there was a decrease in resistances to ciprofloxacin, erythromycin, gentamicin, rifampicin, and tetracycline as compared to MRSA strains collected in an earlier period.

## Additional file

Additional file 1: Table S1. Genetic background and antibiogramms of all 146 MRSA isolates.
