# Supplementary material for: Clonality and antimicrobial susceptibility of methicillin-resistant Staphylococcus aureus at the University Hospital Zurich, Switzerland between 2012 and 2014
Source: Ann Clin Microbiol Antimicrob. 2015 Mar 19;14:14. doi: 10.1186/s12941-015-0075-3 (PMC4369350; doi:10.1186/s12941-015-0075-3)
Supplement: Additional file 1: Table S1. — Genetic background and antibiogramms of all 146 MRSA isolates. [file 12941_2015_75_MOESM1_ESM.docx]

**Additional file 1: Table S1.** Genetic background and antibiogramms of all 146 MRSA isolates.

| **Pred. CC** | **ID** | **Year** | **PFGE-ID** | ***spa*** | **ST** | **CIP** | **C** | **E** | **TE** | **RA** | **GM** | **SXT** |
| --- | --- | --- | --- | --- | --- | --- | --- | --- | --- | --- | --- | --- |
| **CC1** | **USA400** | **Ctrl** | **72d** | **t127** | **1** |  |  |  |  |  |  |  |
|  | 3879 | 2013 | 18 | t127 |  | s | s | s | r | s | s | s |
|  | 3636 | 2012 | 18 | t127 |  | s | s | s | r | s | r | s |
|  | 3948 | 2013 | 18 | t127 |  | s | s | r | s | s | s | s |
|  | 3499 | 2012 | 18 | t127 |  | s | s | s | s | s | s | s |
|  | 3934 | 2013 | 18 | t127 |  | s | s | s | s | s | s | s |
|  | 3957 | 2014 | 18a | t5100 |  | s | s | s | r | s | r | s |
|  | 3569 | 2012 | 72 | t127 | **1** | s | s | r | r | s | s | s |
|  | 3614 | 2012 | 72 | t127 |  | s | u | r | r | s | s | s |
|  | 3795 | 2013 | 72 | t127 |  | s | r | r | r | r | s | s |
|  | 3817 | 2013 | 72 | t127 |  | s | r | r | r | s | s | s |
|  | 3924 | 2013 | 72 | t127 |  | s | u | r | r | s | s | s |
|  | 3702 | 2013 | 72 | t127 |  | s | u | r | r | s | s | s |
|  | 3548 | 2012 | singleton | t127 |  | r | s | r | r | s | s | s |
|  | 3619 | 2012 | singleton | t127 |  | s | s | s | s | s | s | s |
| **CC22** | **EMRSA15 Ctrl** | | **20** | **t032** | **22** |  |  |  |  |  |  |  |
|  | 3498 | 2012 | 20 | t032 |  | r | s | s | s | s | s | s |
|  | 3571 | 2012 | 20 | t032 | **22** | r | s | s | s | s | s | s |
|  | 3635 | 2012 | 20 | t032 |  | r | i | s | s | s | s | s |
|  | 3730 | 2013 | 20 | t032 |  | r | u | r | s | s | s | s |
|  | 3830 | 2013 | 20 | t032 |  | r | s | s | s | s | s | s |
|  | 3864 | 2013 | 20 | t032 |  | r | r | r | s | s | s | s |
|  | 3871 | 2013 | 20 | t032 |  | r | s | s | s | s | s | s |
|  | 3542 | 2012 | 20a | t005 |  | s | s | s | s | s | s | s |
|  | 3929 | 2013 | 20a | t005 |  | s | s | s | s | s | s | s |
|  | 3877 | 2013 | 20b | t025 |  | r | s | s | s | r | r | s |
|  | 3568 | 2012 | 20 | t492 |  | r | s | s | s | s | s | s |
|  | 3865 | 2013 | 20a | t515 |  | r | s | s | s | s | s | s |
|  | 3659 | 2012 | 20c | t852 |  | r | u | r | s | s | r | s |
|  | 3728 | 2013 | 20c | t852 |  | r | u | r | s | s | r | s |
|  | 3794 | 2013 | 20c | t852 |  | r | s | s | s | s | r | s |
|  | 3901 | 2013 | 20c | t852 |  | r | s | s | s | s | r | s |
|  | 3666 | 2012 | 20d | t1771 |  | r | s | s | s | s | s | s |
|  | 3771 | 2013 | 20 | new |  | r | s | s | s | s | s | s |
|  | 3903 | 2013 | 20 | new |  | r | s | s | s | s | s | s |
|  | 3874 | 2013 | 20e | t608 |  | r | s | s | s | s | s | s |
|  | 3617 | 2012 | 63 | t005 | **1327** | s | s | s | s | s | s | s |
|  | 3926 | 2013 | 63 | t005 |  | s | s | s | s | s | s | s |
|  | 3900 | 2013 | 82 | t852 | **22** | r | u | r | s | s | r | s |
|  | 3928 | 2013 | 82a | t4559 |  | r | s | s | s | s | s | s |
| **CC30** | **USA1100 Ctrl** | | **08a** | **t019** | **30** |  |  |  |  |  |  |  |
|  | 3507 | 2012 | 08a | t019 |  | s | s | s | s | s | s | s |
|  | 3547 | 2012 | 08a | t019 | **30** | s | s | s | s | s | s | s |
|  | 3660 | 2012 | 08a | t019 |  | s | s | s | s | s | s | s |
|  | 3945 | 2013 | 08a | t019 |  | s | s | s | s | s | s | s |
|  | 3870 | 2013 | 08a | t318 |  | s | s | s | r | s | s | i |
|  | 3530 | 2012 | 08a | t685 |  | s | s | r | s | s | s | s |
|  | 3774 | 2013 | 08a | t685 |  | s | s | r | s | s | s | s |
|  | 3799 | 2013 | 08a | t685 |  | s | s | r | s | s | s | s |
|  | 3634 | 2012 | 08 | t685 |  | s | s | r | s | s | s | s |
|  | 3970 | 2014 | 08 | t685 |  | s | s | r | s | s | s | s |
|  | 3570 | 2012 | 08 | t318 |  | s | u | r | s | s | s | s |
|  | 3644 | 2012 | 08 | t318 |  | s | s | s | s | s | s | s |
|  | 3797 | 2013 | 08 | t318 |  | s | u | r | s | s | s | s |
|  | 3829 | 2013 | 08 | t318 |  | s | s | s | s | s | s | s |
|  | 3983 | 2014 | 08 | t318 |  | s | u | r | s | s | s | s |
|  | 3868 | 2013 | 08 | t019 |  | s | s | s | s | s | s | s |
| **CC45** | **CHE482** | **Ctrl** | **19** | **t065** | **45** |  |  |  |  |  |  |  |
|  | 3550 | 2012 | 19 | t282 |  | r | s | s | s | s | s | s |
|  | 3719 | 2013 | 19 | t282 | **45** | r | s | s | s | s | s | s |
|  | 3775 | 2013 | 19 | t282 |  | r | s | s | s | s | s | s |
|  | 3742 | 2013 | 19 | t004 |  | s | s | s | s | s | s | r |
|  | 3902 | 2013 | 19 | t015 |  | s | s | s | s | s | s | s |
| **CC5** | **USA100** | **Ctrl** | **21** | **t002** | **5** |  |  |  |  |  |  |  |
|  | 3588 | 2012 | 21a | t002 |  | s | s | s | s | s | s | s |
|  | 3603 | 2012 | 21a | t002 |  | s | s | s | s | s | s | s |
|  | 3610 | 2012 | 21a | t002 |  | s | s | s | s | s | s | s |
|  | 3646 | 2012 | 21a | t002 | **5** | r | u | r | s | s | s | s |
|  | 3689 | 2013 | 21a | t002 |  | s | u | r | s | s | s | s |
|  | 3706 | 2013 | 21a | t002 |  | s | s | s | s | s | s | s |
|  | 3753 | 2013 | 21a | t002 |  | s | u | r | s | s | s | s |
|  | 3754 | 2013 | 21a | t002 |  | r | r | r | s | s | s | r |
|  | 3878 | 2013 | 21a | t002 |  | r | u | r | s | s | s | s |
|  | 3906 | 2013 | 21a | t002 |  | r | r | r | s | s | s | s |
|  | 3911 | 2013 | 21a | t002 |  | r | r | r | s | s | s | s |
|  | 3946 | 2013 | 21a | t002 |  | r | r | r | s | s | s | s |
|  | 3969 | 2014 | 21a | t002 |  | r | r | r | s | s | r | s |
|  | 3486 | 2012 | 21a | t002 |  | r | r | r | s | s | r | s |
|  | 3776 | 2013 | 21b | t003 |  | r | r | r | s | s | s | s |
|  | 3933 | 2013 | 21b | t003 |  | r | r | r | s | s | s | s |
|  | 3942 | 2013 | 21b | t003 |  | r | r | r | s | s | s | s |
|  | 3950 | 2013 | 21b | t003 |  | r | r | r | s | s | s | s |
|  | 3966 | 2014 | 21b | t003 |  | r | u | r | s | s | s | s |
|  | 3567 | 2012 | 21c | t010 |  | s | u | r | s | s | s | s |
|  | 3904 | 2013 | 21c | t010 |  | s | s | s | s | s | r | s |
|  | 3979 | 2014 | 21d | t067 |  | r | r | r | s | s | s | s |
|  | 3585 | 2012 | 21e | t179 |  | r | s | r | s | s | r | s |
|  | 3482 | 2012 | 21e | t179 |  | s | s | s | s | s | s | s |
|  | 3537 | 2012 | 21e | t179 |  | s | s | s | s | s | s | s |
|  | 3521 | 2012 | 21d | t586 |  | r | r | r | s | s | s | s |
|  | 3587 | 2012 | 21f | t311 |  | s | s | s | r | s | s | s |
|  | 3685 | 2013 | 48 | t688 | **5** | s | s | s | r | s | s | s |
|  | 3712 | 2013 | 48 | t688 |  | s | s | s | r | s | s | s |
|  | 3733 | 2013 | 48 | t688 |  | s | s | s | r | s | s | s |
|  | 3736 | 2013 | 48 | t688 |  | s | s | s | r | s | s | s |
|  | 3713 | 2013 | 87 | t002 |  | s | s | s | s | s | s | s |
|  | 3984 | 2014 | 87 | not typable | | r | s | s | s | s | s | s |
| **ST72** | **USA700** |  | **51** | **t148** | **72** |  |  |  |  |  |  |  |
|  | 3642 | 2012 | 51 | t791 |  | s | s | s | s | s | s | s |
|  | 3669 | 2012 | 51 | t791 |  | s | s | s | s | s | s | s |
|  | 3935 | 2013 | 51 | t791 |  | s | s | s | s | s | s | s |
|  | 3586 | 2012 | 51 | t324 |  | s | s | s | s | s | s | s |
|  | 3616 | 2012 | 55 | t791 |  | s | s | r | s | s | s | s |
|  | 3944 | 2013 | 55 | t148 |  | s | s | r | r | s | s | s |
|  | 3816 | 2013 | 55 | t3169 |  | s | s | r | r | s | s | s |
| **CC8** | **USA500** | **Ctrl** | **26** | **t064** | **8** |  |  |  |  |  |  |  |
|  | 3604 | 2012 | 26 | t451 |  | s | s | r | r | s | s | r |
|  | 3502 | 2012 | 26a | t064 |  | r | r | r | s | s | s | r |
|  | 3743 | 2013 | 26b | t967 |  | s | s | s | r | s | s | s |
|  | 3968 | 2014 | 26c | t008 |  | r | r | r | s | s | s | s |
|  | **USA300** | **Ctrl** | **41** | **t008** | **8** |  |  |  |  |  |  |  |
|  | 3503 | 2012 | 41 | t008 | **8** | r | s | r | s | s | s | s |
|  | 3508 | 2012 | 41 | t008 |  | s | s | r | s | s | s | s |
|  | 3513 | 2012 | 41 | t008 |  | r | s | r | s | s | s | s |
|  | 3520 | 2012 | 41 | t008 |  | r | s | r | s | s | s | s |
|  | 3533 | 2012 | 41 | t008 |  | s | s | s | s | s | s | s |
|  | 3594 | 2012 | 41 | t008 |  | r | s | r | r | s | s | s |
|  | 3618 | 2012 | 41 | t008 |  | r | s | r | s | s | s | s |
|  | 3714 | 2013 | 41 | t008 |  | s | s | r | s | s | s | s |
|  | 3793 | 2013 | 41 | t008 |  | r | s | r | s | s | s | s |
|  | 3875 | 2013 | 41 | t008 |  | s | s | r | s | s | s | s |
|  | 3910 | 2013 | 41 | t008 |  | r | r | r | s | s | s | s |
|  | 3943 | 2014 | 41 | t008 |  | s | s | s | s | s | r | s |
|  | 3956 | 2014 | 41 | t008 |  | r | s | r | s | s | s | s |
|  | 3627 | 2012 | 41 | t008 |  | r | s | s | s | s | s | s |
|  | 3867 | 2013 | 41b | t197 |  | r | s | r | s | s | s | s |
|  | 3912 | 2013 | 49 | t304 |  | s | s | s | s | s | s | s |
|  | 3481 | 2012 | 49 | t008 |  | s | s | s | s | s | s | s |
|  | 3729 | 2013 | 49 | t008 |  | s | s | s | s | s | s | s |
|  | 3744 | 2013 | 49 | t008 |  | s | s | s | s | s | s | s |
|  | 3873 | 2013 | 49 | t008 |  | s | s | s | s | s | s | s |
|  | 3905 | 2013 | 49 | t008 |  | s | u | r | r | s | s | s |
|  | 3913 | 2013 | 49 | t008 | **8** | s | s | s | s | s | s | s |
| **ST239** | 3500 | 2012 | 11 | t969 |  | r | s | r | r | r | r | r |
|  | **3908** | 2013 | 81 | t037 | **239** | r | r | r | r | i | r | s |
|  | 3982 | 2014 | 81 | t037 |  | r | r | r | r | r | r | s |
|  | 3673 | 2012 | singleton | t030 |  | r | r | r | r | r | r | s |
| **CC88** | 3608 | 2012 | 77 | t448 |  | s | s | s | s | s | s | s |
|  | 3538 | 2012 | 84 | t730 |  | s | u | r | r | s | s | s |
|  | 3672 | 2012 | 84a | t786 |  | s | s | s | r | s | s | s |
|  | 3876 | 2013 | 84 | t786 | **88** | s | u | r | s | s | s | r |
|  | 3670 | 2012 | 25 | new |  | s | s | s | s | s | s | s |
|  | 3731 | 2013 | singleton | t355 |  | s | s | s | s | s | r | s |
|  | 3907 | 2013 | 25 | t454 |  | s | s | s | s | s | r | s |
| ST6 | 3777 | 2013 | 54 | t304 | **6** | s | s | s | s | s | s | s |
| ST6 | 3976 | 2014 | 54 | t304 |  | s | s | s | s | s | s | s |
|  | 3798 | 2013 | 75 | t044 |  | s | s | s | r | s | s | s |
|  | 3557 | 2012 | singleton | t437 |  | s | r | r | r | s | s | s |
|  | 3947 | 2013 | singleton | t189 |  | r | r | r | s | s | r | s |
|  | 3752 | 2013 | singleton | t267 |  | s | s | s | s | s | r | s |
|  | 3800 | 2013 | singleton | t1028 |  | s | s | s | s | s | r | s |
|  | 3751 | 2013 | singleton | t2453 |  | r | u | r | r | s | s | s |
|  | 3620 | 2012 | 62 | t657 |  | r | s | r | s | s | r | s |
|  | 3687 | 2013 | not typable | t1885 |  | r | r | r | s | r | s | r |

ST, Sequence type; CIP, Ciprofloxacin; C, Clindamycin; E, Eryhtromycin; TE, Tetracyclin; RA, Rifampicin; GM, Gentamicin; SXT, Sulfamethoxazole/trimethoprim; s, susceptible; r, resistant; i, intermediate resistance; u, inducible resistance.
